# Non-Targeted UHPLC-Q-TOF/MS-Based Metabolomics Reveals a Metabolic Shift from Glucose to Glutamine in CPB Cells during ISKNV Infection Cycle

**DOI:** 10.3390/metabo9090174

**Published:** 2019-09-04

**Authors:** Xiaozhe Fu, Xixi Guo, Shiwei Wu, Qiang Lin, Lihui Liu, Hongru Liang, Yinjie Niu, Ningqiu Li

**Affiliations:** 1 Pearl River Fisheries Research Institute, Chinese Academy of Fishery Sciences, Key Laboratory of Fishery Drug Development, Ministry of Agriculture and Rural Affairs, Key Laboratory of Aquatic Animal Immune Technology, Guangzhou 510380, China (X.F.) (X.G.) (S.W.) (Q.L.) (L.L.) (H.L.) (Y.N.); 2 College of Fisheries and Life Science, Shanghai Ocean University, Shanghai 201306, China

**Keywords:** ISKNV, metabolomics profile, differential metabolites, glucose metabolism, glutamine metabolism

## Abstract

Infectious spleen and kidney necrosis virus (ISKNV) has caused serious economic losses in the cultured mandarin fish (*Siniperca chuatsi*) industry in China. Host metabolism alteration induced by disease infection may be the core problem of pathogenesis. However, to date, little is known about the disease-induced fish metabolism changes. In this study, we first reported ISKNV, the fish virus, induced metabolism alteration. The metabolomics profiles of Chinese perch brain cells (CPB) post-ISKNV infection at progressive time points were analyzed using the UHPLC-Q-TOF/MS technique. A total of 98 differential metabolites were identified. In the samples harvested at 24 hours post-infection (hpi; the early stage of ISKNV infection), 49 differential metabolites were identified comparing with control cells, including 31 up-regulated and 18 down-regulated metabolites. And in the samples harvested at 72 hpi (the late stage of ISKNV infection), 49 differential metabolites were identified comparing with control cells, including 27 up-regulated and 22 down-regulated metabolites. These differential metabolites were involved in many pathways related with viral pathogenesis. Further analysis on the major differential metabolites related to glucose metabolism and amino acid metabolism revealed that both glucose metabolism and glutamine metabolism were altered and a metabolic shift was determined from glucose to glutamine during ISKNV infection cycle. In ISKNV-infected cells, CPB cells prefer to utilize glucose for ISKNV replication at the early stage of infection, while they prefer to utilize glutamine to synthetize lipid for ISKNV maturation at the late stage of infection. These findings may improve the understanding of the interaction between ISKNV and host, as well as provide a new insight for elucidating the ISKNV pathogenic mechanism.

## 1. Introduction

Infectious kidney and spleen necrosis virus (ISKNV), an important viral pathogen of mandarin fish, is the type species of the genus Megalocytivirus, the family Iridoviridae [1]. ISKNV disease has caused great economic losses for the cultured mandarin fish (also known as Chinese perch) in China during the past few decades [2,3]. Because of its wide host range and high mortality for different fish species, ISKNV has been identified as one of the most important causative agents of fish and listed by the International Epizootic Office (OIE) [4,5,6]. Until now, which cell type is susceptible to ISKNV infection has been not clear, and only two fish cell lines susceptible to ISKNV infection have been developed all over the world; that is, MFF cell line from mandarin fish fry cell and CPB cell line from mandarin fish brain [6,7]. Although the brain is not the target organ, ISKNV can be detected in the diseased fish brain. At present, the transcriptome and proteome of CPB cells infected with ISKNV have been reported [8,9]. Several studies have also investigated the interaction between ISKNV and hosts [4,8,9,10,11,12,13,14,15]. However, the ISKNV pathogenesis has not been fully understood to date.

Metabolomics is the comprehensive study of metabolic reactions. It represents the global assessment of metabolites in a biological sample and reports the closest information to the phenotype of the biological system under certain study. Thus, metabolomics studies frequently state that metabolome is a closer reflection of the phenotype of an organism, tissue, or cell than the other “omics” analyses of proteomics, transcriptomics, and genomics [16,17,18,19]. At present, nuclear magnetic resonance (NMR) spectroscopy and mass spectrometry (MS) have emerged as the most frequently used analytical platforms. However, given the limitations of NMR in terms of sensitivity, MS coupled to liquid chromatography (LC), gas chromatography (GC), or capillary electrophoresis (CE) has emerged as a powerful technological alternative [17]. Typically, MS-based metabolomics encompasses two main approaches: The “targeted analysis”, in which dozens to hundreds of metabolites are analyzed, and the “untargeted analysis”, in which all the possible metabolites are unbiasedly analyzed in a given biological sample [18]. Because non-targeted metabolomics can reflect the total metabolites information to the greatest extent, it has been widely used to reveal multiple dynamic responses of metabolites under different conditions [16]. At present, owing to its high sensitivity and specificity, ultra-high-performance liquid chromatography/quadrupole-time-of-flight-mass spectrometry (UHPLC-Q-TOF/MS) has been widely used to determine metabolites to investigate interaction between pathogen and host. Several studies have been reported concerning the metabolite changes of the host cells during virus infection [20,21,22,23]. However, for aquatic animal viruses, only one paper has been published on the metabolomics changes of shrimp hemocytes post- white spot syndrome virus (WSSV) infection [24]. Metabolomics has enabled new insights into viral pathogenesis targeting by metabolic shift, as well as antiviral treatment strategies. However, the metabolites of mandarin fish involved in ISKNV infection is not available.

In the previous study, we have identified the early stage (24 hours post-infection (hpi)) and the late stage (72 hpi) of ISKNV infection [9]. To determine the metabolic profiles of CPB cells infected with ISKNV at different progressive times, we conducted an untargeted metabolomics analysis to characterize the alterations at the ISKNV-infected cells compared to the non-infected cells using UHPLC-Q-TOF/MS. A number of differential metabolites were identified between the ISKNV-infected groups and the control groups at 24 and 72 hpi. These differential metabolites were involved in many pathways of viral pathogenesis. A more comprehensive method and better comprehension of the interaction between ISKNV and host metabolic shift will shed new light on the prevention of ISKNV disease. This is the first report on the fish disease-induced metabolomics to date.

## 2. Results

### 2.1. Quality Control of Untargeted Metabolomics Analysis

**Figure 1:**
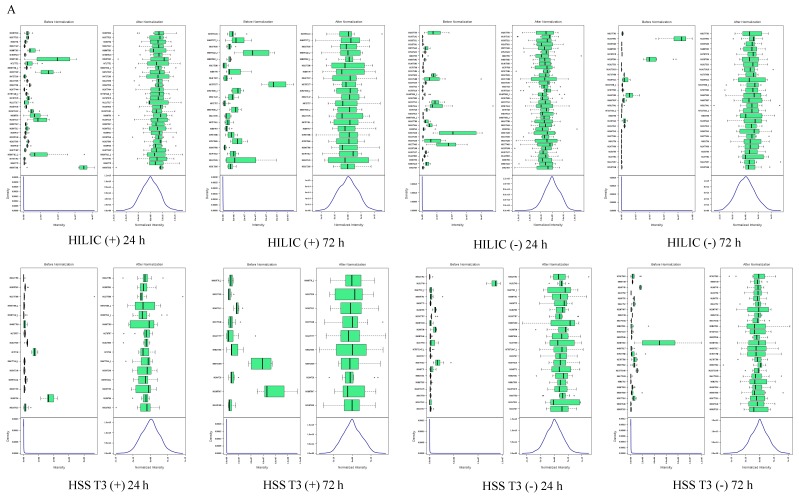

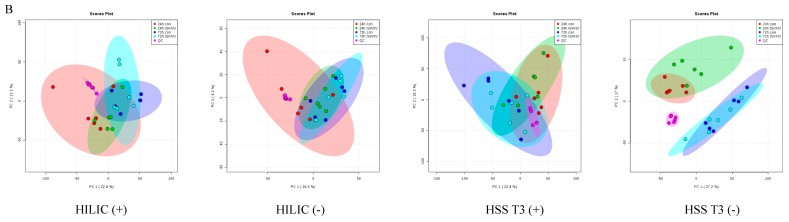
Quality assessment of UHPLC-Q-TOF/MS metabolomics data in CPB cells. (**A**) The distribution of input data values before (left) and after (right) normalization; (**B**) The PCA score plots of *Siniperca chuatsi* cells sample. Chromatographic columns: ACQUITY BEH Amide 1.7 µm [HILIC] and ACQUITY HSS T3 1.8 µm [HSS T3]. Abbreviations: (+) positive ion modes; (−) negative ion modes.

For the untargeted metabolomics analysis, the data were subjected to a data integrity check, and no missing values were detected. After log transformation and Pareto scaling to normalize the data, the metabolomics data presented a normal distribution after these processes (Figure 1A). Principal component analysis (PCA) was carried out using the molecular features of all the groups from the study, including QC samples. Figure 1B shows that QC injections (pink) were clustered tightly in PCA space (Figure 1B), which indicates the reliability and satisfactory reproducibility of the metabolic profiling during the experiment. The check for outliers by PCA on the single group of samples is shown in supplementary material (Figure S1). A total of 39,158 molecular features were extracted, in which 12,846 molecular features were from HILIC [ACQUITY BEH Amide 1.7 µm] and 26,312 molecular features from HSS T3 [ACQUITY HSS T3 1.8 µm], respectively (Table 1).

### 2.2. Identification of Differential Metabolites from ISKNV-Infected CPB Cells

**Figure 2:**
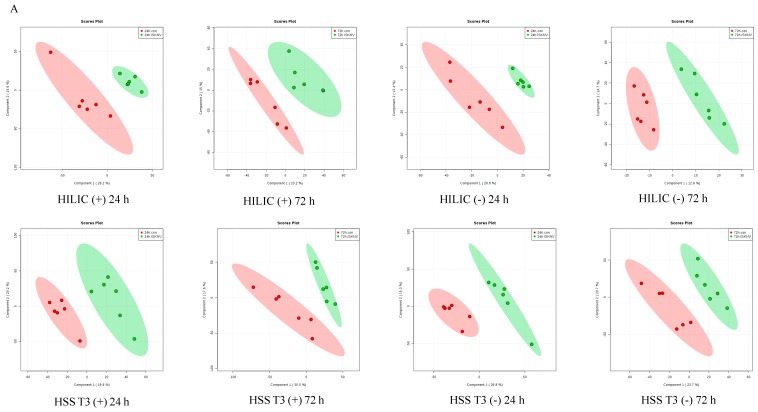

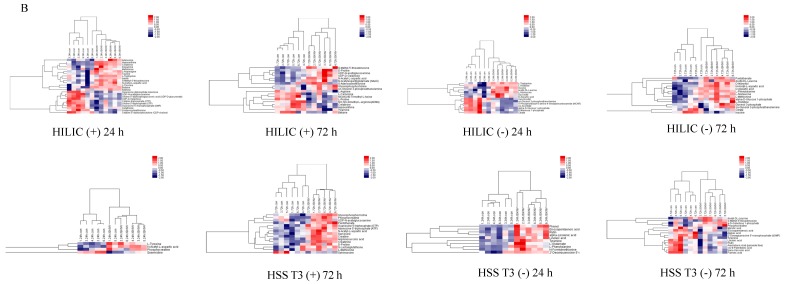
Analysis of differential metabolites in ISKNV-infected CPB cells. (**A**) PLS-DA plots based on the UHPLC-Q-TOF/MS data from the ISKNV groups vs. the control groups; (**B**) Heat map of clustering analysis of the ISKNV groups vs. the control groups.

A supervised PLS-DA model was established to identify ion peaks of differential metabolite profiles between ISKNV-infected cells and control cells. As shown in Figure 2A, we observed clear separation between the ISKNV-infected cells and control cells. The model evaluation parameters (R2, Q2) obtained with 10 cycles of cross-validation of all PLS-DA models are shown in Table 2. When VIP > 1 and *p* < 0.05, metabolites were identified as the significant differential metabolites. When VIP > 1 and 0.05 < *p* < 0.1, metabolites were identified as the differential metabolites. Based on the VIP calculated by the PLS-DA model, a heat map of hierarchical clustering analysis is shown in Figure 2B. Results of PLS-DA analysis and the heat map show that the metabolite abundance profiles were similar in the parallel groups.

A total of 98 differential metabolites were determined between ISKNV-infected cells and control cells. In the samples harvested at 24 hpi (the early stage of ISKNV infection), 49 differential metabolites were identified comparing with control cells, including 31 up-regulated and 18 down-regulated metabolites. In the samples harvested at 72 hpi (the late stage of ISKNV infection), 49 differential metabolites were identified comparing with control cells, including 27 up-regulated and 22 down-regulated metabolites. The differential metabolite components are described in Table 3.

### 2.3. Functional Annotation and Enrichment Analysis of Differential Metabolites

**Figure 3:**
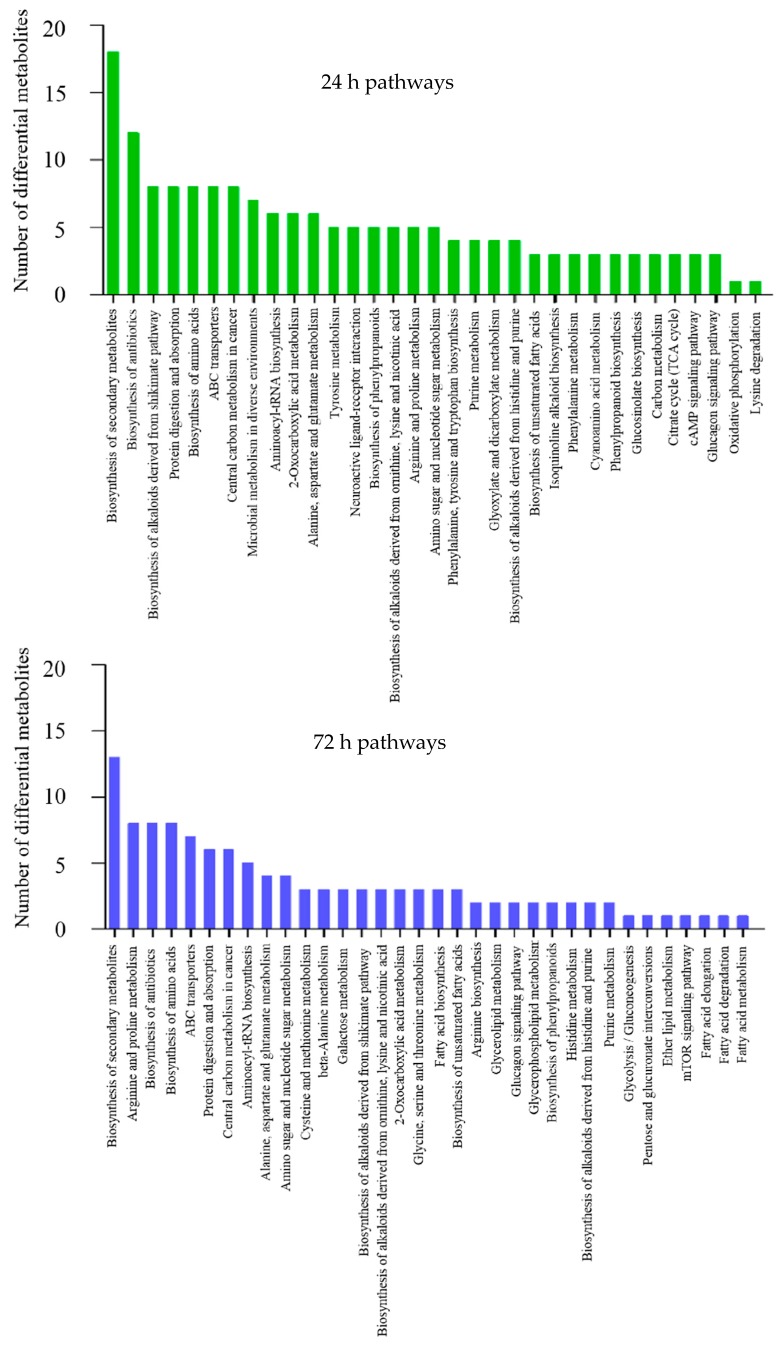
KEGG enrichment analysis for differential metabolites upon ISKNV infection.

To understand the functions of differential metabolites and the biological processes related to ISKNV infection, all differential metabolites were mapped to terms in the KEGG database. KEGG enrichment analysis showed that these differential metabolites were mainly enriched in the following seven metabolic pathways at 24 hpi: Biosynthesis of secondary metabolites, biosynthesis of antibiotics, biosynthesis of alkaloids derived from the shikimate pathway, protein digestion and absorption, biosynthesis of amino acids, ABC transporters, and central carbon metabolism in cancer. These differential metabolites were mainly enriched in the following five metabolic pathways at 72 hpi: Biosynthesis of secondary metabolites, arginine and proline metabolism, biosynthesis of antibiotics, biosynthesis of amino acids, and ABC transporters. The metabolic pathways enriched by differential metabolites are described in Figure 3. In this study, we focused particularly on a limited number of important host pathways, including glucose metabolism and amino acid metabolism.

### 2.4. Analysis of Differential Metabolites Related to Glucose Metabolism

**Figure 4:**
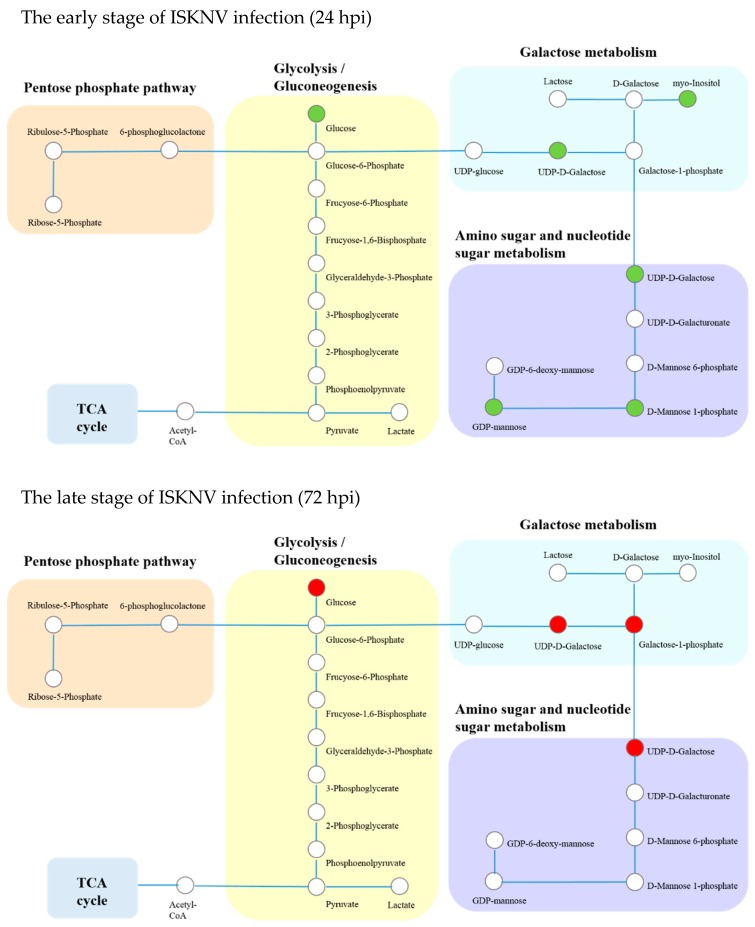
Integrated networks of ISKNV infection outlining biochemical pathways related to glycolysis/gluconeogenesis, the pentose phosphate pathway, galactose metabolism, and amino sugar and nucleotide sugar metabolism. Metabolites are represented by circles. Changes in the levels of metabolites (circles) relative to the control groups are color-coded to represent up-regulation (red) or down-regulation (green). Colorless boxes indicate that no difference was detected. Metabolomics data were collected from six groups of parallel samples of CPB cells using UHPLC-Q-TOF/MS. Abbreviations: hpi, hours post-infection.

Differential metabolites related to glucose metabolism, including glycolysis/gluconeogenesis, galactose metabolism, the pentose phosphate pathway, and the amino sugar and nucleotide sugar metabolism pathways, are shown in Table 3. The changes in glucose metabolism at 24 and 72 hpi are shown in Figure 4. Our results show that these differential metabolites were mainly enriched in the glycolysis/gluconeogenesis, galactose metabolism, and the amino sugar and nucleotide sugar metabolism pathways. As shown in Figure 4, compared to control cells, glucose concentration of ISKNV-infected cells was significantly reduced at 24 hpi, but significantly elevated at 72 hpi, which inferred that ISKNV mainly utilized glucose in its early stage of infection (24 hpi) but not in its late stage of infection (72 hpi).

**Figure 5:**
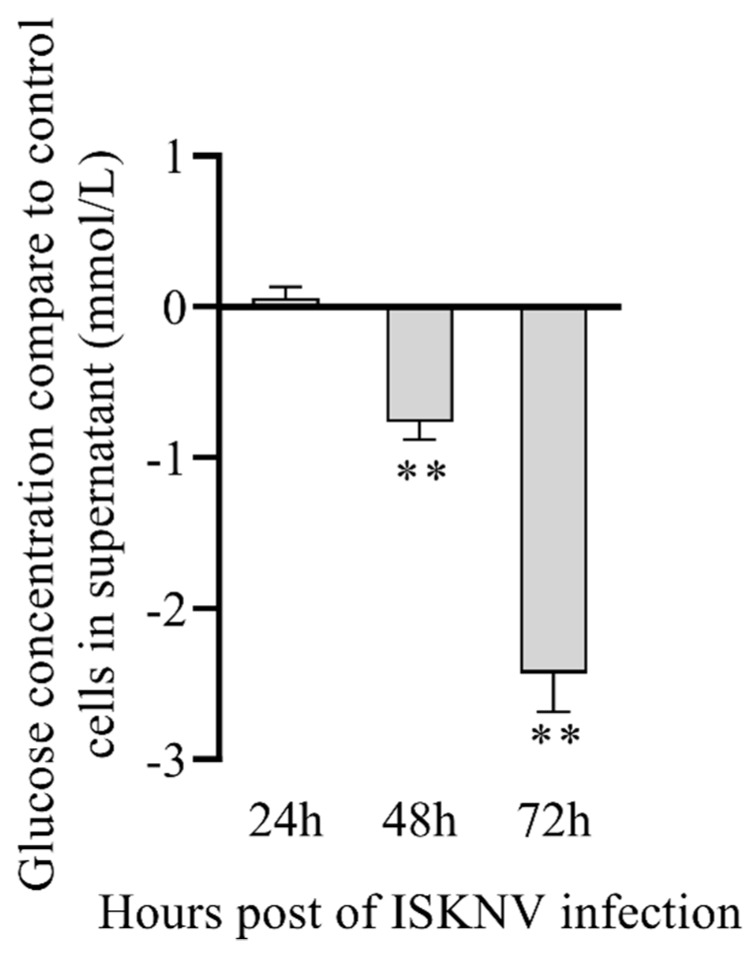
The comparative changes of glucose concentrations in cell culture supernatants of the ISKNV-infected group and the control group. The comparative change between ISKNV-infected cells and control cells is defined as C [glucose concentration in supernatant _(ISKNV-infected cells)_] − C [glucose concentration in supernatant _(control cells)_].

To further validate the metabolomics result, we firstly detected the viability of CPB cells in the absence of glucose. We observed that CPB cells could not survive more than 48 h in glucose-free culture medium, which indicates that glucose is required for CPB cells (data not shown). Then, we detected the glucose concentration in supernatant of the ISKNV infection group compared to the control group. Figure 5 shows that glucose concentration in culture medium of the ISKNV infection group was higher than in the control group at 24 hpi (no significant difference), and lower than that in the control group at 48 and 72 hpi (significant difference), which indicates that ISKNV replication mainly consumed the intracellular glucose at 24 hpi, then glucose was absorbed and accumulated into CPB cells for the next round of progeny virus infection at 72 hpi. This result is consistent with the metabolomics result. All of the above results show that ISKNV multiplication in CPB cells needs glucose, and mainly utilizes glucose in the early stage of virus infection (24 hpi) but not in the late stage of virus infection (72 hpi).

### 2.5. Analysis of Differential Metabolites Related to Amino Acid Metabolism

**Figure 6:**
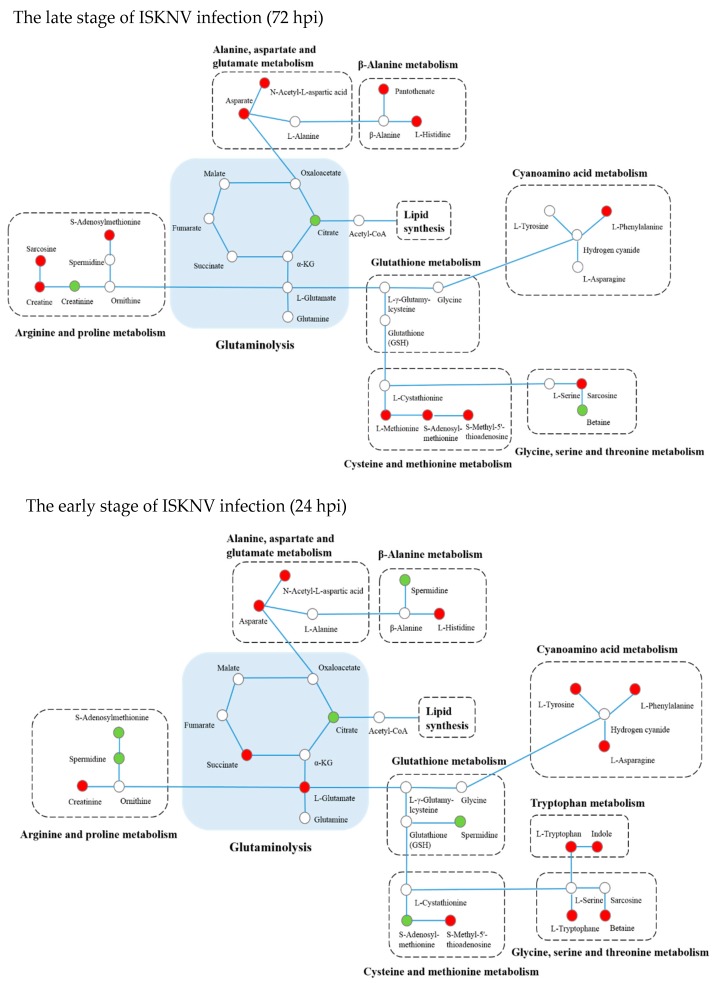
Integrated networks of ISKNV infection outlining biochemical pathways related to amino acid metabolism. Metabolites are represented by circles. Changes in the levels of metabolites (circles) relative to control groups are color-coded to represent up-regulation (red) or down-regulation (green). Colorless boxes indicate that no difference was detected. Metabolomics data were collected from six parallel samples of CPB cells using UHPLC-Q-TOF/MS.

Differential metabolites related to amino acid metabolism, including glutaminolysis, arginine and proline metabolism, Alanine, aspartate and glutamate metabolism, β-Alanine metabolism, glutathione metabolism, cysteine and methionine metabolism, glycine, serine and threonine metabolism, tryptophan metabolism, and cyanoamino acid metabolism pathways, are shown in Table 3. The changes in amino acid metabolism at 24 and 72 hpi are shown in Figure 6, mainly enriched in the glutaminolysis, β-Alanine metabolism, alanine, aspartate and glutamate metabolism, arginine and proline metabolism, glutathione metabolism, cysteine and methionine metabolism, cyanoamino acid metabolism, tryptophan metabolism and glycine, and serine and threonine metabolism pathways.

**Figure 7:**
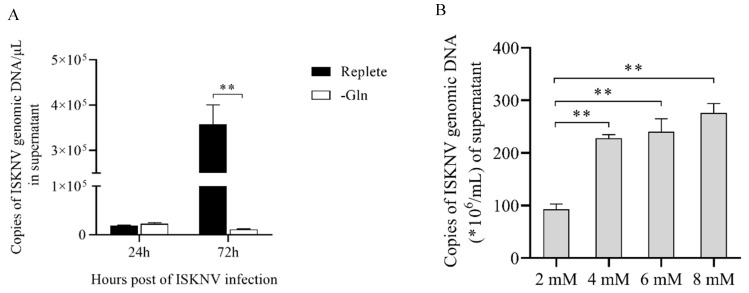
Glutamine increases the yield of ISKNV in CPB cells. The viral copy number was determined by fluorescent real-time PCR (TaqMan). (**A**) The yield of ISKNV in culture medium with 2 mM glutamine (Replete) or without glutamine (–Gln); (**B**) the yield of ISKNV in culture medium supplement with 2, 4, 6, and 8 mM glutamine at 7 days post-infection (dpi).

It has been reported that glutamine is required for some virus replication [25]. Thus, in this section, we mainly focused on the glutamine metabolism. As shown in Figure 6, glutamate concentration was significantly elevated in ISKNV-infected cells compared to control cells at 24 hpi, but could not be detected at 72 hpi, which indicates that ISKNV multiplication increased the uptake of glutamine at the early ISKNV replication stage, and increased consumption of glutamine at the late ISKNV replication stage. To further precisely determine the role of glutamine for ISKNV multiplication, the ISKNV yield was measured by real-time quantitative PCR (qPCR). As shown in Figure 7A, the ISKNV yield in replete medium was similar to that in the –Gln group at 24 hpi, but significantly increased at 72 hpi. Furthermore, ISKNV yield at 7 days post-infection (dpi) was increased in a dose-dependent manner with culture medium supplemented with 2, 4, 6, and 8 mM glutamine (Figure 7B). The above results illustrate that ISKNV multiplication in CPB cells mainly relies on glutamine at its late stage of infection.

Citrate, as an intermediate metabolite of glutamine metabolism, is involved in the TCA cycle. The metabolomics results show that citrate concentration was decreased at 24 and 72 hpi, which illustrates that ISKNV multiplication consumed citrate. In our previous study, we found that the ISKNV yield was increased when the culture medium was supplemented with citrate in a dose-dependent manner [26]. Thus, citrate may be an important metabolite for ISKNV multiplication.

**Figure 8:**
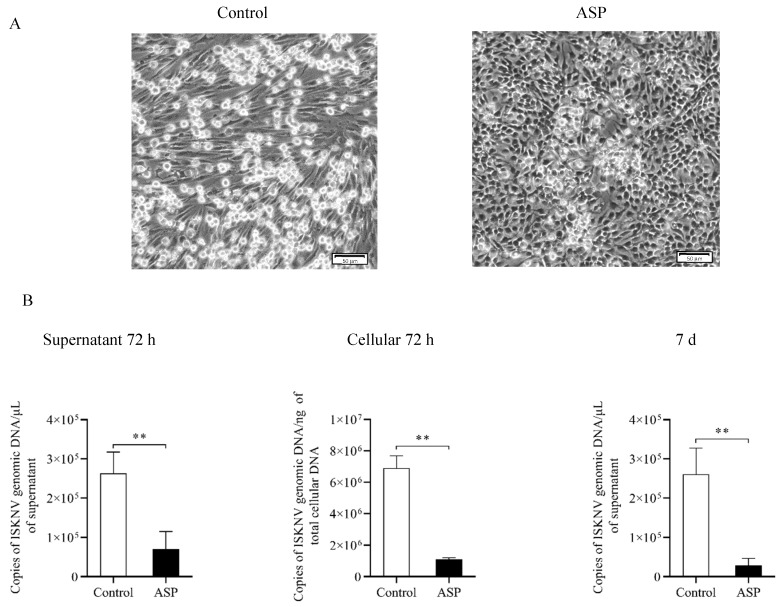
Aspartate inhibits the yield of ISKNV in CPB cells. (**A**) CPE of ISKNV-infected CPB cells (scale bar = 50 μm); (**B**) the viral copy number was validated via fluorescent real-time PCR (TaqMan).

It has been reported that carbon for aspartate (ASP) synthesis is supplied by glutamine [27,28,29]. Interestingly, we found that D-Aspartic acid was accumulated at different stages of ISKNV replication. In order to investigate this appearance, the addition of asparate was added to the ISKNV culture medium. The result of CPE and viral copy number shows that the addition of ASP inhibited ISKNV replication (Figure 8), which suggests that it also plays an important role in the ISKNV multiplication.

## 3. Discussion

Metabolomics is a powerful tool for studying metabolic processes, identifying crucial biomarkers responsible for metabolic characteristics, and revealing metabolic mechanisms. Many studies have also shown that pathogens can cause changes to host metabolomes, which involve glucose metabolism, nucleotide metabolism, amino acid metabolism, and lipid metabolism, to adapt to their new environment, such as hepatitis C virus (HCV), WSSV, and dengue virus (DENV) [20,23,30]. The current investigation identified metabolic alteration in ISKNV-infected cells and results showed that CPB cells prefer to utilize glucose at the early stage of ISKNV infection (24 hpi), while they prefer to utilize glutamine at the late stage of ISKNV infection (72 hpi).

Viruses rely on host cellular metabolism to facilitate their multiplication. Glucose, as an important carbon source in the host cells metabolism, is catalyzed into pyruvate through many steps, and its metabolism is modified dramatically during viral infection [31,32,33,34]. The previous study found that the first infection cycle of ISKNV started from 0 to 72 hpi, and according to the replication kinetics of ISKNV in CPB cells, 24 and 72 hpi were regarded as the early and the late stages of ISKNV replication, respectively [9]. Our previous proteomic profile results showed that ISKNV replication enhanced glucose metabolism in CPB cells in early-stage infection (24 hpi) [9]. In the present study, metabolomics results also show that ISKNV mainly utilized glucose in its early stage of infection (24 hpi), but not in its late stage of infection (72 hpi). However, interestingly, there was no difference at 24 hpi between the two groups by detecting the glucose concentration in supernatant. We presumed that the glucose starvation experiment was not done before the ISKNV infection because CPB cells could not survive more than 48h, which led to glucose storage in the CPB cells. Thus, ISKNV replication mainly consumed the glucose stored in the cells and not the extracellular glucose at 24 hpi, which caused no difference in the glucose concentration of culture medium at 24 hpi. It has been reported that many viruses were found altering glucose metabolism in host cells during their infection, such as hepatitis B virus (HBV) [31], Kaposi’s sarcoma herpesvirus (KSHV) [32], DENV [33], and HCV [34]. Glucose is usually involved in the glycolysis, PPP, and TCA cycle pathways, which is primarily utilized for TCA cycle during standard growth conditions, but mainly utilized for glycolysis in most cancer cells or some viral-infected cells [24]. It also has been reported that WSSV infection induces the Warburg Effect via the PI3K-Akt-mTOR pathway [24]. However, whether Warburg Effect is triggered during ISKNV infection or not requires further validation.

Glutamine serves as a primary carbon donor for the TCA cycle, which is initially oxidized to glutamate in a reaction catalyzed by glutaminase. Thereafter, glutamate dehydrogenase (GDH) catalyzes the conversion of glutamate to α-ketoglutarate (α-KG), which enters the tricarboxylic acid (TCA) cycle to provide a variety of important TCA intermediates. Glutamine plays an important role in the infection and replication of human immunodeficiency virus (HIV) [35], human cytomegalovirus (HCMV) [36], and vaccinia virus (VACA) [37]. It has also been demonstrated that ISKNV infection alters glutamine metabolism, and glutamine is required for ISKNV efficient multiplication in CPB cells [26]. In the present study, we found that glutamine is required for ISKNV at its late stage of infection, and the ISKNV yield was increased when the culture medium was supplemented with glutamine in a dose-dependent manner. Citrate is a kind of intermediate metabolite of glutamine involved in TCA cycle. In this paper, we found that citrate concentration of the ISKNV infection group was decreased at 24 and 72 hpi. Interestingly, some cancer cells in hypoxia prefer to use glutamine, not glucose, to convert into citrate for lipid synthesis [38], and some cancer cells mainly use citrate that is generated by carboxylate glutamine to lipogenesis [39,40,41]. Specifically, fatty acid metabolism mainly occurs in the late stage of ISKNV infection (Figure 3). The above results illustrate that ISKNV prefers to utilize glutamine at the late stage of infection, and citrate, generated by carboxylate glutamine, contributes to lipogenesis for ISKNV maturation in CPB cells, which needs to be further explored.

Aspartate can be produced from glutamine via both reductive and oxidative pathways [42], and is the synthetic precursor of various amino acids, purine, and pyrimidine bases. It plays an important role in cell proliferation. Aspartate is also a restricted amino acid in the proliferation of tumor cells and more studies have shown that the inhibition of aspartate synthesis leads to tumor cell apoptosis [27,43,44,45,46]. Its most important role is to regulate the respiration of mitochondria and to participate in the production of ATP through a malate–aspartate shuttle system [47]. It was reported that the malate–aspartate shuttle system can be supplemented with exogenous aspartate to recover cell respiration [43]. In the present study, the finding of elevated aspartate in the ISKNV-infected cells and its inhibitory role for ISKNV multiplication suggests that aspartate may contribute to maintaining the vitality of CPB cells, which we plan to investigate in future studies.

## 4. Materials and Methods

### 4.1. Cells and Viruses

Chinese perch brain cell line (CPB) was established and stored in our lab [6]. CPB cells were cultured in DMEM high glucose medium (Gibco, USA) supplemented with 10% fetal bovine serum (FBS) (Gibco, USA) at 28 °C. ISKNV-QY was isolated and stored in our lab previously [6]. ISKNV was propagated in CPB cells fed DMEM high glucose medium containing 2% FBS at 28 °C and its titer was determined by TCID_50_ assay. The virus was stocked at −80 °C until use.

### 4.2. Sample Acquisition

A total of four group cells were collected, including ISKNV-infected cells groups (24 and 72 hpi) and negative control cells groups (24 and 72 hpi). Briefly, CPB cells were infected with ISKNV (MOI of 1.0) or cultured with medium (negative control). After 2 h inoculation and adsorption at 28 °C, the inoculum was removed and the cells were washed twice with PBS before adding DMEM high glucose medium (containing 2% FBS). Then ISKNV-infected cells and negative control cells were harvested at 24 and 72 hpi, respectively. Before the samples were collected, the cells were washed with pre-cooled PBS twice, and washed with pre-cooled 0.9% NaCl once. The wash solution was discarded completely, and 1 mL methanol/acetonitrile/water (2:2:1, v/v) was added. Then, the cells were collected with scraper to a centrifuge tube. At each time point, six parallel samples (2 tubes per sample) were collected as biological replicates. The cells number of each sample was 8 × 10^6^ cells/mL. The samples were stored at −80 °C prior to further processing for UHPLC-Q-TOF/MS analysis.

### 4.3. Sample Preparation

Before analysis, the samples were thawed at 4 °C. Then, the tubes were vortexed for 30 s, ultrasound for 10 min, and centrifuged for 15 min (14,000 g, 4 °C). The supernatants were dried with vacuum, and then the samples were stored at −80 °C until the UHPLC-Q-TOF/MS analysis. Lyophilized samples were re-dissolved in 100 μL solution buffer containing acetonitrile/water (1:1, v/v). The samples were vortexed and centrifuged for 15 min (14,000 g, 4 °C). The supernatants were used for UHPLC-Q-TOF/MS analysis. The quality control (QC) samples were prepared and were analyzed together with the other samples to determine the instrument state and equilibrium chromatography–mass spectrometry system before injection, and to evaluate the stability of the system during the whole experiment.

### 4.4. UHPLC-Q-TOF/MS Analysis

Metabolic profiling of CPB cells samples was performed on an Agilent 1290 Infinity LC system (Agilent Technologies, Santa-Clara, California, USA) combined with an AB SCIEX Triple TOF 6600 System (AB SCIEX, Framingham, MA, USA) [48]. Chromatographic separation was performed on ACQUITY BEH Amide 1.7 µm (2.1 × 100 mm) [HILIC] and ACQUITY HSS T3 1.8 µm (2.1 × 100 mm) [HSS T3] columns for both positive and negative models. The column was maintained at 25 °C, and 2 μL samples were injected into a column, with the delivery flow rate of 300 μL/min. The mobile phase of HILIC, consisting of A (water + 25 mM ammonium acetate + 25 mM ammonia) and B (acetonitrile), was used. The elution gradient initially started from 85% B for 1 min, linearly reduced to 65% B at 12 min, linearly reduced to 40% B at 12.1 min for 2.9 min, and then returned to 85% B for approximately 5 min of equilibrium. The mobile phase of HSS T3, consisting of A (0.1% formic acid in water) and B (0.1% formic acid in acetonitrile), were used in positive ionization mode, while C (0.5 mM ammonium fluoride in water) and D (acetonitrile) were used in negative ionization mode. In the positive (negative) model, the elution gradient initially started with 1% B (D) for 1.5 min, linearly increased to 99% B (D) at 13 min, maintained for 3.5 min, and then returned to 1% B (D) for about 3.5 min of equilibrium.

TOF/MS was performed on both (positive and negative) ionization modes. The electrospray ionization (ESI) source conditions separated by HILIC chromatography were described as Gao et al. [49]. The ESI source conditions separated by HSS T3 chromatography were set as follows: Ion source gas 1, 40 psi; ion source gas 2, 80 psi; curtain gas, 30 psi; source temperature, 650 °C; ion spray voltage floating, 5000 V (+) and −5000 V (−). The other conditions were similar to the HILIC chromatography. The analysis process was conducted with the assistance of Applied Protein Technology Co., Ltd. (Shanghai, China).

### 4.5. Data Analysis

The raw data were converted into mzML format files using the Proteo Wizard MS converter tool and then processed using XCMS online software (https://xcmsonline.scripps.edu/landing_page.php?pgcontent=mainPage) for doing peak alignment, retention time correction, and peak area extraction. Metabolite structural identification was performed by comparing the accuracy of *m/z* values (< 25 ppm) and matching of second stage spectra with the laboratory’s self-built database (Applied Protein Technology Co., Ltd., Shanghai, China). MetaboAnalyst 4.0 (http://www.metaboanalyst.ca) was employed for further statistical analysis [50].

Multivariate data analyses, including the unsupervised principal component analysis (PCA), supervised partial-least squares discrimination analysis (PLS-DA), and single dimensional statistical analysis, were performed. PLS-DA models were validated based on the multiple correlation coefficient (R2) and cross-validated R2 (Q2) in cross-validation and permutation tests by applying 2000 iterations (*p* > 0.001). The variable importance in the projection (VIP) value (> 1) in the PLS-DA model and Student’s *t*-test were applied to measure the significance of differential metabolites. A *p*-value less than 0.05 was deemed statistically significant.

Based on the differential metabolites identified by comparing ISKNV-infected groups and control groups, Kyoto Encyclopedia of Genes and Genomes (KEGG) pathway (http://www.genome.jp/kegg/) analysis was conducted to investigate the metabolomics pathways affected by ISKNV infection. Briefly, the enrichment level of each metabolomics pathway was calculated using Fisher’s exact test, and a *p*-value less than 0.05 was considered statistically significant.

### 4.6. Determination of Glucose Consumption in Supernatant

CPB cells were infected with ISKNV (MOI of 1.0) for 2 h. Subsequently, cells were washed twice with PBS and fed DMEM high glucose (Gibco, USA) containing 2% FBS. Culture supernatants of ISKNV-infected cells and negative control cells at various hours post-infection (24, 48, and 72 hpi) were harvested. Glucose consumption was measured by a High Sensitivity Glucose Assay Kit (Sigma).

### 4.7. L-Glutamine and D-Asparate Replenishing Experiment

CPB cells were infected with ISKNV (MOI of 1.0) for 2 h, and then washed twice with PBS. For the L-Glutamine replenishing experiment, cells were cultured with glutamine-free DMEM (–Gln) or replete DMEM (supplemented with 2, 4, 6, or 8 mM additional glutamine). For the D-Asparatic acid replenishing experiment, cells were cultured with DMEM (control) or DMEM supplemented with the additional 8 mM asparate (ASP). The cytopathic effect (CPE) of different groups was observed. The ISKNV copies of different groups were determined by real-time quantitative PCR (qPCR) as per a previous report [51].

## 5. Conclusions

All of above, we first reported the metabolomics profiles of CPB cells post-ISKNV infection at 24 and 72 hpi. A total of 98 differential metabolites were identified. Metabolic shift alterations in ISKNV-infected cells show that CPB cells prefer to utilize glucose for ISKNV replication at the early stage of infection (24 hpi), while they prefer to utilize glutamine to synthetize lipid and maintain cell viability for ISKNV maturation at the early stage of infection (72 hpi). This is the first report on the metabolomics changes induced by virus infection. This study may provide new insight into the viral pathogenesis.

## Figures and Tables

**Table 1:** The number of features.

Detection Mode	HILIC	HSS T3
Positive ion	8408	13,027
Negative ion	4438	13,285
Total	12,846	26,312

**Table 2:** The evaluation parameters of the PLS-DA model.

Detection Mode	HILIC			HSS T3		
	Number of PC	R2 (cum)	Q2 (cum)	Number of PC	R2 (cum)	Q2 (cum)
Positive ion	3	0.9811	0.58625	4	0.99866	0.60266
Negative ion	4	0.99984	0.49266	5	0.91667	0.6841

**Table 3:** List of differential metabolites in the ISKNV infection groups compared to the control groups.

Time	Name	VIP	Fold Change	*p*-Value
**24 h**	Eicosapentaenoic acid	2.535	6.723	0.0058 *
	Phenol	1.850	4.428	0.0203 *
	Rutin	2.019	4.307	0.0137 *
	Tyramine	2.083	4.233	0.0041 *
	L-Phenylalanine	1.783	3.481	0.0529
	Hypoxanthine	2.320	2.949	0.0000 *
	L-Glutamate	1.154	2.721	0.0750
	Creatinine	2.120	2.470	0.0000 *
	N-Formylmethionine	1.299	2.272	0.0111 *
	2′-Deoxyguanosine 5′-monophosphate (dGMP)	1.786	2.217	0.0361 *
	alpha-Linolenic acid	1.134	2.114	0.0164 *
	L-Tryptophan	1.677	2.102	0.0149 *
		1.795	1.923	0.0023 *
	Phosphocreatine	1.106	2.100	0.0914
	Inosine	1.930	2.003	0.0010 *
	Linoleic acid	1.155	1.974	0.0030 *
	Succinate	1.745	1.939	0.0112 *
	L-Tyrosine	1.608	1.904	0.0082 *
		1.870	3.430	0.0472 *
	Adenosine	1.478	1.800	0.0144 *
	L-Asparagine	1.448	1.775	0.0229 *
	Betaine	1.574	1.772	0.0287 *
	L-Histidine	1.906	1.657	0.0573
	Acetyl-DL-Leucine	1.691	1.643	0.0014 *
	L-Norleucine	1.670	1.624	0.0006 *
	S-Methyl-5′-thioadenosine	1.387	1.606	0.0008 *
	Dopamine	1.272	1.605	0.0326 *
	Taurine	1.268	1.582	0.0323 *
	Indole	1.236	1.536	0.0630
	Adenine	1.053	1.423	0.0313 *
	Maltotriose	1.065	1.417	0.0365 *
	N-Acetyl-L-aspartic acid	1.084	1.369	0.0036 *
		1.833	2.434	0.0000 *
	D-Aspartic acid	1.117	1.291	0.0359 *
	Spermidine	1.217	0.786	0.0345 *
	GDP-mannose	1.097	0.735	0.0220 *
	UDP-N-acetylglucosamine	1.300	0.667	0.0072 *
	Cytidine triphosphate (CTP)	1.127	0.666	0.0835
	Uridine 5′-diphosphoglucuronic acid (UDP-D-glucuronate)	1.372	0.646	0.0052 *
	Uridine 5′-triphosphate (UTP)	1.252	0.639	0.0365 *
	myo-Inositol	1.204	0.636	0.0001 *
	sn-Glycerol 3-phosphoethanolamine	1.285	0.607	0.0003 *
	UDP-D-Galactose	1.573	0.592	0.0012 *
	Guanosine 5′-monophosphate (GMP)	1.325	0.567	0.0386 *
	5′-Phosphoribosyl-5-amino-4-imidazolecarboxamide (AICAR)	1.044	0.561	0.0481 *
	alpha-D-Glucose 1-phosphate	1.301	0.529	0.0181 *
	D-Mannose 1-phosphate	1.188	0.517	0.0252 *
	S-Adenosylmethionine	1.795	0.488	0.0046 *
	cis-Aconitate	1.115	0.473	0.0913
	Cytidine 5′-diphosphocholine (CDP-choline)	1.997	0.392	0.0057 *
	Citrate	1.463	0.371	0.0649
	Gutathione	1.980	0.314	0.0326 *
**72 h**	D-Mannitol	2.611	2.182	0.0021 *
	S-Methyl-5′-thioadenosine	2.143	2.135	0.0142 *
		1.627	1.411	0.0817
	Guanosine 5′-triphosphate (GTP)	2.066	2.072	0.0011 *
	D-Proline	2.028	2.051	0.0324 *
		1.230	1.175	0.0232 *
	N-Acetyl-L-aspartic acid	2.262	1.946	0.0008 *
		1.923	1.550	0.0005 *
		1.955	1.854	0.0036 *
	Pantothenate	2.257	1.868	0.0202 *
		1.252	1.211	0.0070 *
	UDP-N-acetylglucosamine	1.970	1.709	0.0059 *
		1.419	1.230	0.0936
	S-Adenosylmethionine	1.548	1.593	0.0596
	UDP-D-Galactose	1.728	1.561	0.0164 *
	S-Lactoylglutathione	1.620	1.555	0.0847
	Adenosine 5′-triphosphate (ATP)	1.586	1.490	0.0008 *
	Acetyl-DL-Leucine	1.370	1.452	0.0458 *
		1.214	1.257	0.0707
	N-Acetylaspartylglutamate (NAAG)	1.426	1.450	0.0657
	Argininosuccinic acid	1.558	1.376	0.0000 *
	alpha-D-Glucose 1-phosphate	1.358	1.337	0.0391 *
	Glycerol 3-phosphate	1.190	1.325	0.0802
	Glycerophosphocholine	1.210	1.300	0.0383 *
		1.823	1.514	0.0039 *
	L-Phenylalanine	1.353	1.273	0.0042 *
	L-Methionine	1.264	1.271	0.0062 *
		1.100	1.144	0.0715
	L-Histidine	1.208	1.267	0.0592
	D-Aspartic acid	1.207	1.264	0.0298 *
	sn-Glycerol 3-phosphoethanolamine	1.143	1.254	0.0494 *
		1.086	1.221	0.0983
	L-Norleucine	1.209	1.227	0.0267 *
	Phosphocreatine	1.343	1.215	0.0646
		1.097	1.157	0.0302 *
	Creatine	1.239	1.212	0.0010 *
	a-D-Galactose 1-phosphate	1.188	1.169	0.0061 *
	Sarcosine	1.178	1.168	0.0024 *
	L-Arginine	1.098	0.784	0.0895
	L-Carnosine	1.010	0.776	0.0912
	N6,N6,N6-Trimethyl-L-lysine	1.368	0.729	0.0064 *
	Citrate	1.517	0.711	0.0980
	2′-Deoxyguanosine 5′-monophosphate (dGMP)	1.160	0.703	0.0022 *
	L-Proline	1.421	0.686	0.0194 *
	Myristic acid	1.088	0.684	0.0370 *
	Azelaic acid	1.131	0.682	0.0401 *
	Inosine	1.903	0.635	0.0021 *
	NG,NG-dimethyl-L-arginine(ADMA)	1.691	0.630	0.0046 *
	Creatinine	1.846	0.582	0.0149 *
		1.258	1.186	0.0012 *
	Tyramine	2.186	0.567	0.0860
		2.556	0.325	0.0102 *
	Eicosapentaenoic acid	1.496	0.552	0.0913
	Linoleic acid	1.519	0.540	0.0937
	Betaine	1.490	0.540	0.0626
	cis-9-Palmitoleic acid	2.072	0.473	0.0205 *
	Rutin	1.965	0.437	0.0737
	Arachidonic Acid (peroxide free)	1.858	0.432	0.0791
	trans-Vaccenic acid	1.908	0.418	0.0600
	Hypoxanthine	2.683	0.377	0.0025 *
	Palmitic acid	2.329	0.347	0.0214 *
	Sphingosine	2.057	0.178	0.0890

Abbreviations: VIP, variance importance for projection; * *P* < 0.05.

## Supplementary Materials

The following are available online at https://www.mdpi.com/2218-1989/9/9/174/s1, Figure S1: the check for outliers by PCA on the single group samples.
