# Supplementary material for: Non-Targeted UHPLC-Q-TOF/MS-Based Metabolomics Reveals a Metabolic Shift from Glucose to Glutamine in CPB Cells during ISKNV Infection Cycle
Source: Metabolites. 2019 Sep 4;9(9):174. doi: 10.3390/metabo9090174 (PMC6780522; doi:10.3390/metabo9090174)
Supplement: Supplementary file 1 [file metabolites-09-00174-s001.pdf]

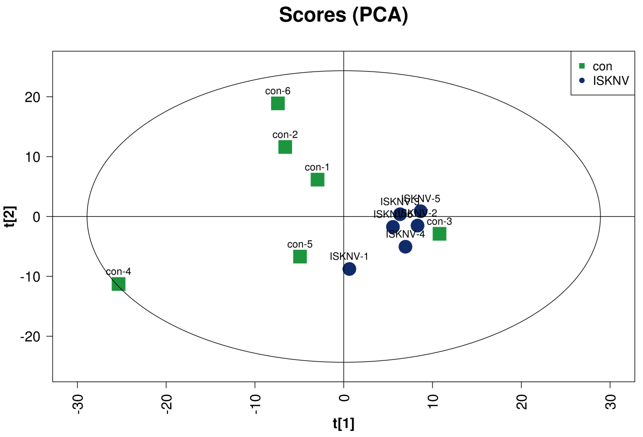

HILIC (+) 24h

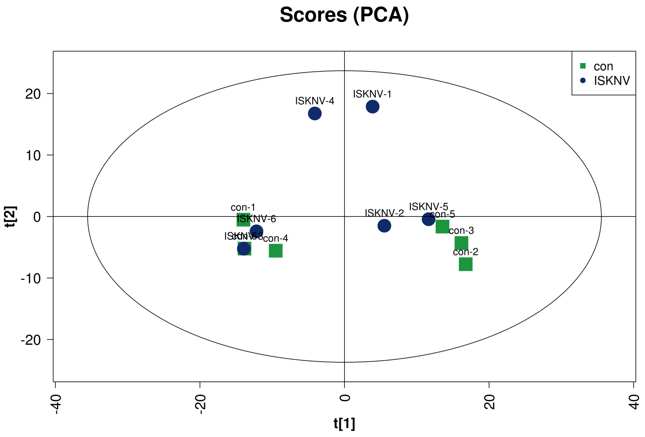

HILIC (+) 72h

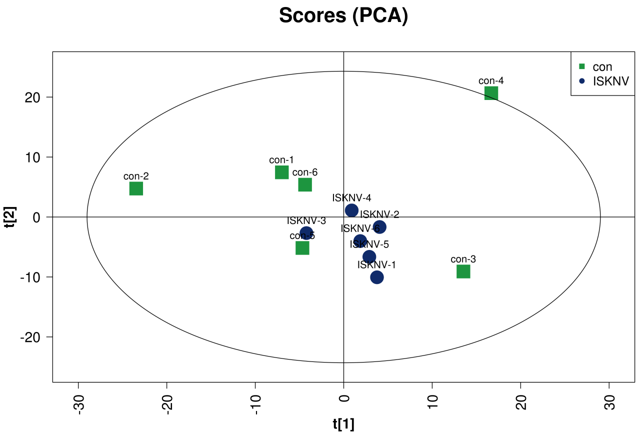

HILIC (-) 24h

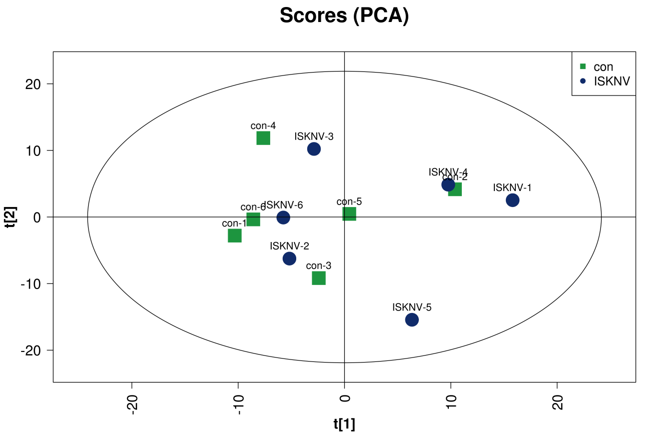

HILIC (-) 72h

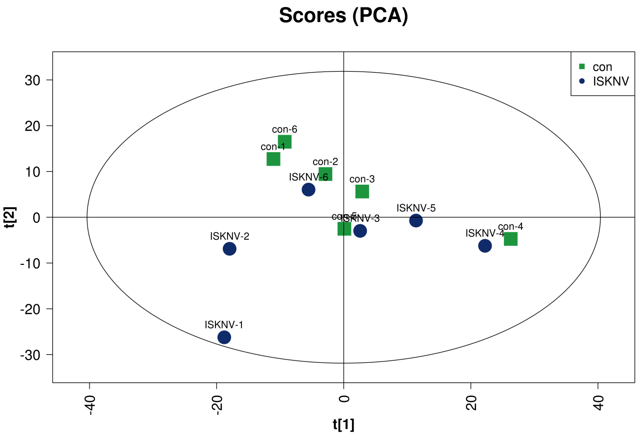

HSS T3 (+) 24h

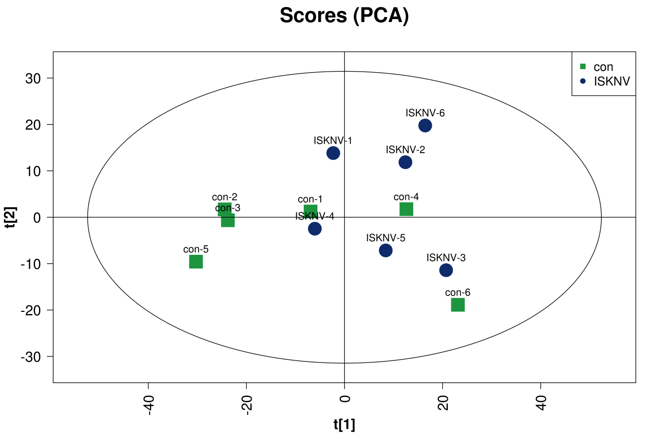

HSS T3 (+) 72h

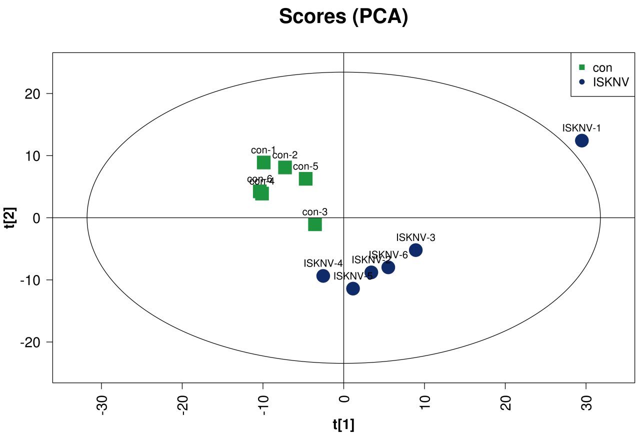

HSS T3 (-) 24h

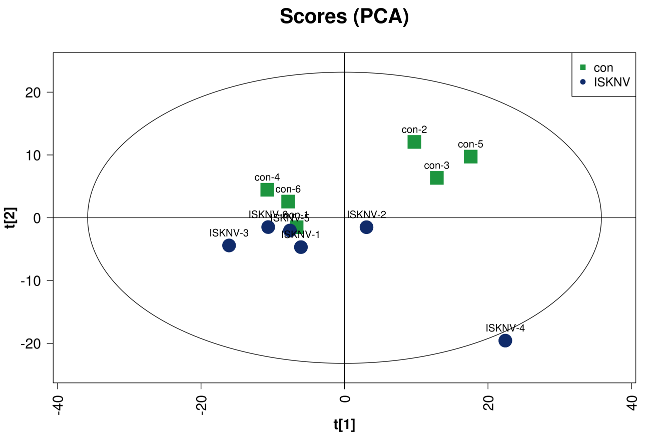

HSS T3 (-) 72h

Figure. S1 The check for outliers by PCA on the single group samples

Chromatographic columns: ACQUITY BEH Amide 1.7 μm [HILIC] and ACQUITY HSS T3 1.8 μm [HSS T3].

Abbreviations: (+): positive ion modes; (-) negative ion modes
